# Long noncoding RNA TCONS_00026334 is involved in suppressing the progression of colorectal cancer by regulating miR‑548n/TP53INP1 signaling pathway

**DOI:** 10.1002/cam4.3473

**Published:** 2020-09-28

**Authors:** Mingming Zhu, Yang Luo, Antao Xu, Xitao Xu, Ming Zhong, Zhihua Ran

**Affiliations:** ^1^ Division of Gastroenterology and Hepatology Key Laboratory of Gastroenterology and Hepatology Ministry of Health Shanghai Inflammatory Bowel Disease Research Center Renji Hospital School of Medicine Shanghai Jiao Tong University, Shanghai Institute of Digestive Disease Shanghai China; ^2^ Department of Gastrointestinal Surgery Ren Ji Hospital School of Medicine Shanghai Jiao Tong University Shanghai China

## Abstract

Recently, long noncoding RNAs (lncRNAs) were recognized as significant therapeutic targets in tumors. Our previous microarray analysis showed that lncRNA *TCONS_000026334* expression was reduced in metastatic colorectal cancer (CRC) tissues. The objective of this study was to research the biological functions of *TCONS_000026334* and the potential mechanism during the development of CRC. *TCONS_00026334* transcription levels were detected in CRC tissues from 86 patients and different CRC cell lines. The clinical prognosis factors related to TCONS_00026334 expression were then analyzed. *TCONS_000026334* was overexpressed from plasmid pcDNA3.1‐TCONS_ 000026334 or knocked down using a small interfering RNA (siRNA). Furthermore, bioinformatics approach and luciferase reporter gene assays were utilized to search for candidate miRNAs of TCONS_00026334 and identify the downstream target genes. The results indicated that *TCONS_00026334* expression in 86 CRC tissues was markedly lower than that in non‐cancerous tissues. The aberrant expression of *TCONS_00026334* correlated negatively with larger tumor size, distant metastasis, serological carcinoembryonic antigen level, and unfavorable survival of patients with CRC. *TCONS_00026334* overexpression could inhibit the aggressive phenotypes of CRC in *vitro* and in *vivo*. Conversely, *TCONS_00026334* silencing accelerated CRC cell proliferation and invasion. We then verified that *TCONS_00026334* upregulated the expression level of *TP53INP1*, a target gene of miR‐548n, via direct binding to miR‐548n as a competing endogenous RNA. Taken together, our study showed that *TCONS_00026334* acts as an anti‐tumor and anti‐metastatic gene by regulating the miR548n/*TP53INP1* axis in the development of CRC.

## Graphical abstract

Our study showed that lncRNA TCONS_00026334 acts as an anti‐tumor and anti‐metastatic gene by regulating the miR548n/TP53INP1 axis in the development of colorectal cancer.

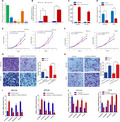

## 1 INTRODUCTION

Globally, the burden of colorectal cancer (CRC) is increasing.^1^, ^2^ Due to distant metastasis and relapse, the prognosis of patients with CRC is still very poor.^3^, ^4^ In order to improve the early diagnosis of CRC and promote targeted therapy, it is necessary to understand in more detail the molecular mechanism of the initiation and progression of CRC.^5^, ^6^

Long noncoding RNAs (lncRNAs) are transcribed RNAs comprising more than 200 nucleotides without the potential of encoding proteins. LncRNAs play an important role in regulating of the occurrence, invasion, and metastasis of malignant tumors; therefore, they might represent a new group of therapeutic targets and cancer biomarkers.^7^, ^8^, ^9^ Several studies have shown that aberrant expression or mutations of various lncRNAs, including *MALAT1*, *GAPLINC*, *NEAT1*, and *CCAT2*, enhance the metastasis, invasion, and proliferation of CRC cells.^10^, ^11^, ^12^, ^13^ However, many lncRNAs are still being discovered and are yet to be annotated, and the mechanisms involving lncRNAs in the complex progression of CRC invasion require further study.^14^ In our previous investigation, transcriptome microarray analysis was conducted to explore potential differentially expressed lncRNAs from clinical tissues samples of patients with CRC with or without distant metastasis. Among the 79 differentially expressed lncRNAs, *TCONS_00026334* was obviously downregulated in CRC with stage IV distant metastasis compared with that in tissues without distant metastasis, which indicated that *TCONS_00026334* might play a vital role in the progression of CRC as an anti‐metastatic gene. *TCONS_00026334* is a 2463‐bp sense‐overlapping lncRNA located at 18q21.2.^15^ (Supplementary Information TCONS_00026334). Therefore, lncRNA *TCONS_00026334* was selected as the candidate lncRNA to the study the invasion and metastasis of CRC.

As short noncoding RNAs, microRNAs (miRNAs) modulate protein‐coding genes and are involved in the pathogenesis of several diseases, including human cancers.^16^ Recently, interesting interactions between lncRNAs and miRNAs have been recognized, and emerging evidence suggests that this crosstalk has a significant impact on the cancer metastasis cascade.^17^, ^18^ However, the molecular mechanism of the interaction between TCONS_00026334 and miRNAs in CRC is still unclear. In the current study, further bioinformatics tools and luciferase reporter gene assays were utilized to search for candidate miRNAs of *TCONS_000026334* and identify the downstream target genes. The bioinformatics analysis suggested the existence of miR‐548n binding sites in *TCONS_00026334* and *TP53INP1*, which serves as an anti‐oncogene in CRC.^19^ Therefore, *TP53INP1* was screened out as a novel candidate of miR‐548n. Furthermore, we verified that *TCONS_000026334* acts as ceRNAs by regulating the expression of TP53INP1 through sponge miR‐548n to suppress the progression of CRC.

## 2 MATERIALS AND METHODS

### 2.1 Patients and sample collection

Eighty‐six snap‐frozen CRC samples and paired samples of peritumoral tissues were obtained from Renji Hospital between January 2013 and January 2015, with informed consent of patients and approval by the institutional ethics committee. The recruited patients had not yet received preoperative chemotherapy and/or radiation therapy. The fresh para‐tumorous tissues were sampled at 2–3 cm distal to the tumor and non‐cancerous tissues were sampled at least 5 cm distal to the tumor; all tissues were subjected to histological examination. Tumor tissues from distant metastases were obtained from 17 of the 86 patients. The specimens were collected and placed immediately at −80°C until protein or RNA was extracted.

### 2.2 Cell lines and cell culture

All human CRC cell lines Caco2, SW1116, HCT116, lovo, and HT29 cells, were obtained from the Cell Bank of Chinese Academy of Sciences (Shanghai, China). FHC (human fetal colon cells) was supplied by American Type Culture Collection (ATCC). All the cell lines were cultured at 37°C in a humidified atmosphere containing 5% CO_2_ in RPMI (Roswell Park Memorial Institute) 1640 medium from Invitrogen (Carlsbad), which contained 10% FBS (fetal bovine serum; Gibco) and 1% streptomycin/penicillin (Sigma‐Aldrich).

### 2.3 RNA extraction and qRT‐PCR

The total RNA was extracted from cell lines and tissues using the Trizol reagent (Invitrogen), according to the manufacturer's protocol. While nuclear and cytoplasmic RNA was extracted from cultured cells using the PARIS kit (Life Technologies) according to the manufacturer's instructions. A Reverse Transcription Kit was purchased from Takara (Shiga, Japan) for reverse transcribing, and qRT‐PCR. For lncRNA quantification, the internal control comprised mRNA encoding glyceraldehyde‐3‐phosphate dehydrogenase (GAPDH). The PCR primers for the present study are summarized in Table S1. The 2(−2Delta Delta CT) method was performed to calculate the relative expression level of lncRNA.^20^

### 2.4 Western blotting analysis

Whole cells or tissues were lysed with RIPA lysis buffer, and protein concentrations were quantified by a BCA Protein Assay kit (Pierce Biotechnology). The separation of proteins was completed by 10% SDS‐PAGE (loaded at 30 µg/lane) and then electroblotted onto a PVDF membrane (Millipore). Incubation with 5% nonfat milk was used to block non‐specific binding to the membranes. Next, the membranes were incubated with the primary antibodies at 4°C overnight and then HRP‐conjugated secondary antibodies at room temperature for one hour. Primary antibodies against TP53INP1, β‐actin, and P21 were purchased from Abcam. ECL Western blotting system was utilized to scan the immunoreactive protein.

### 2.5 Proliferation assay

A CCK8 assay kit (Dojindo) was performed to measure cell viability. The 3000 cells were seeded into each well of 96‐well plates and then incubated with medium containing 1% FBS. Subsequently, each well was added ten microliters of CCK‐8 reagent at specified time intervals, and then incubated for 2 h. The cell number was measured using the absorbance at 450 nm, recorded using a scanning spectrophotometer with a microplate reader.

### 2.6 Transwell assay

Corning BioCoat Matrigel Invasion Chambers (Corning Costar Corp) were used to perform the cell invasion assay in triplicate. We added 1 × 10^5^ cells to per insert in the upper chamber, and 200 ml of supplemented medium was added to the bottom chamber. After incubating for 24 h, the cells were fixed with 4% paraformaldehyde on the filter surface, followed by staining using hematoxylin and eosin. Under an optical microscope, five regions were randomly selected in each chamber to count the cells.

### 2.7 SiRNA and plasmid transfection

Oligonucleotides, including the *TCONS_00026334* short interfering RNA (siRNA), miR‐548n mimics, *TP53INP1* siRNA, and corresponding negative controls, were all synthesized from the GenePharma (Shanghai, China). The *TCONS_00026334* cDNA was cloned into the pcDNA3.1 expression vector. GenePharma constructed the *TCONS_00026334* cDNA, *TP53INP1* cDNA, and the respective negative controls. 0.5 × 10^5^ cells/ml of SW1116 and HCT116 cells were seeded into six‐well plates and subsequently incubated for 24 h before being transfected with the above‐mentioned oligonucleotides and plasmids with Lipofectamine 2000 (Invitrogen).

### 2.8 Luciferase assays

The online software program miRDB (http://www.mirdb.org) was applied to predict the relationship between *TCONS_00026334* and miR‐548n, and between *TP53INP1* and miR‐548n. Predicted wild type (WT) and mutant (MUT) binding sites for miR‐548n in the 3′‐untranslated region (UTR) of *TCONS_00026334* and *TP53INP1* mRNA, which were named as pmirGLO‐TCONS_00026334‐WT or pmirGLO‐TCONS_00026334‐MUT, pmirGLO‐TP53INP1‐WT or pmirGLO‐ TP53INP1‐MUT, were cloned into the luciferase reporter vector pmirGLO (Gene array Biotechnology). SW1116 and HCT116 cells that had reached 70% confluence and had been subjected to 1 hour or serum starvation, then 5 × 10^4^ cells were plated into each well of 12‐well plates. Subsequently, 0.5 μg of WT or MUT *TCONS_00026334* plasmids and 25 pmol of miR‐548n mimic or control were used to co‐transfect the cells. The cells were harvested after being transfected for 48 h, and then analyzed using a Dual Luciferase Assay system (Promega). Triplicate experiments were performed.

### 2.9 Tumor xenograft model experiment

The local ethics committee of Renji Hospital approved the mouse experiments. This study used BALB/c nude mice under female‐specific pathogen free (SPF) conditions with the approval of Animal Care and Use Committee of Renji Hospital. Briefly, 5 × 10^6^ cells were subcutaneously injected into the armpits of nude mice every three days. Tumor growth was monitored and subcutaneous tumors were measured since initial injection, and then were euthanized at 25 days. Next, the tumors were removed, weighed, photographed, and fixed in the 4% paraformaldehyde for 24 h. Eventually, xenograft specimens were stored at −80°C for subsequent experiments after being frozen in liquid nitrogen.

### 2.10 Statistical analysis

All experiments were repeated at least three times and reported as mean ± SD. Student's *t*‐test and chi‐squared test were requested to analyze the statistical differences. The Kaplan–Meier estimator was applied to measure the survival profiles, which were analyzed with the log rank test. These statistical analyses were conducted by SPSS Statistics 22.0 software (IBM Corp.). Statistical significance was accepted at *p* < 0.05.

## 3 RESULTS

### 3.1 Poor prognosis of CRC was associated with *TCONS_00026334* expression

**Figure 1:**
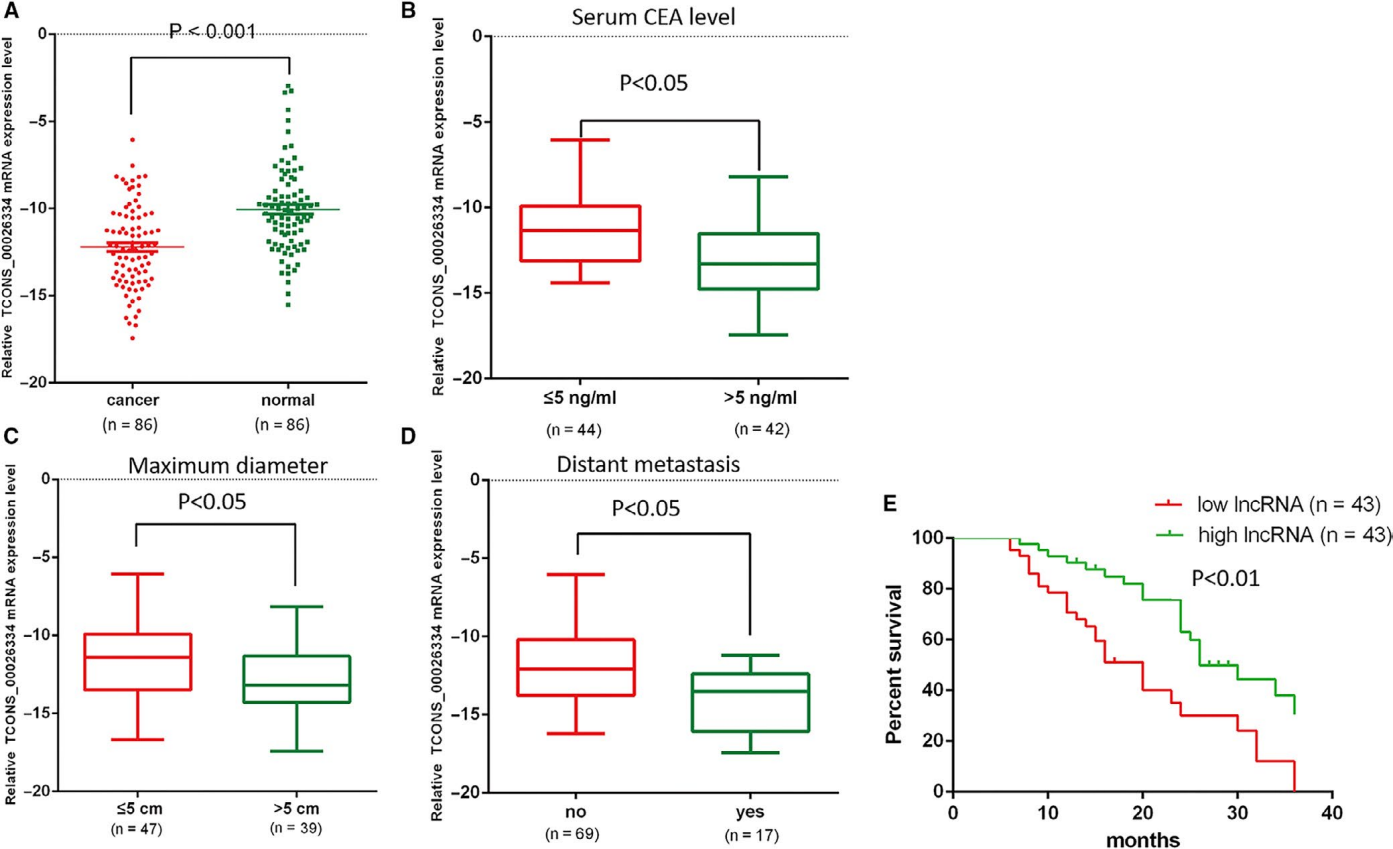
Relative expression level of TCONS_00026334 in the CRC tissues and its relationship with clinical features of patients with CRC. (A) QRT‐PCR data demonstrating the significant downregulation of TCONS_00026334 in CRC tissues by comparison to that in adjacent non‐cancerous tissues. (B–D) The correlation between TCONS_00026334 expression and serum CEA level, maximum tumor diameter, and distant metastasis in patients with CRC. (E) Overall survival comparisons between patients with low and high TCONS_00026334 expression.

The transcript abundance of *TCONS_00026334* was measured in 86 pairs of CRC tissues and matched normal adjacent tissues to determine the expression level of *TCONS_00026334* in CRC. The results showed that the expression level of *TCONS_00026334* was downregulated in tumor tissues of patients with CRC in comparison to that in noncancerous tissues (Figure 1A). Then, the possible correlation between *TCONS_00026334* and clinical characteristics of patients with CRC was investigated. The data shown in Figure 1(B–D) indicated that a low *TCONS_00026334* level was correlated with maximum tumor diameter (*p* < 0.01), distant metastasis (*p* < 0.001), and the serum CEA level (*p* < 0.01).

Then, based on the median expression value of *TCONS_00026334*, patients with CRC were subdivided into relative low expression group and relative high expression group. Kaplan–Meier analyses indicated that patients in the relative high *TCONS_00026334* expression group had significantly longer overall survival (OS) by comparison with that in the relative low expression group (*p* = 0.001, Figure 1E). Positive associations between the presence of distant metastasis (M), clinical stage, CEA level, and tumor size and the levels of *TCONS_00026334* were observed (Table S2). However, there was no association between the *TCONS_00026334* level other factors, including age, gender, and tumor location. Next, multivariate analysis was performed to assess the relationship between prognosis and *TCONS_00026334* expression. The results showed that in patients with CRC, *TCONS_00026334* expression and the presence of distant metastasis in CRC tissues were independent predictors of OS (Table S3).

### 3.2 TCONS_00026334 inhibits CRC cells proliferation and invasion *in vitro*

**Figure 2:**
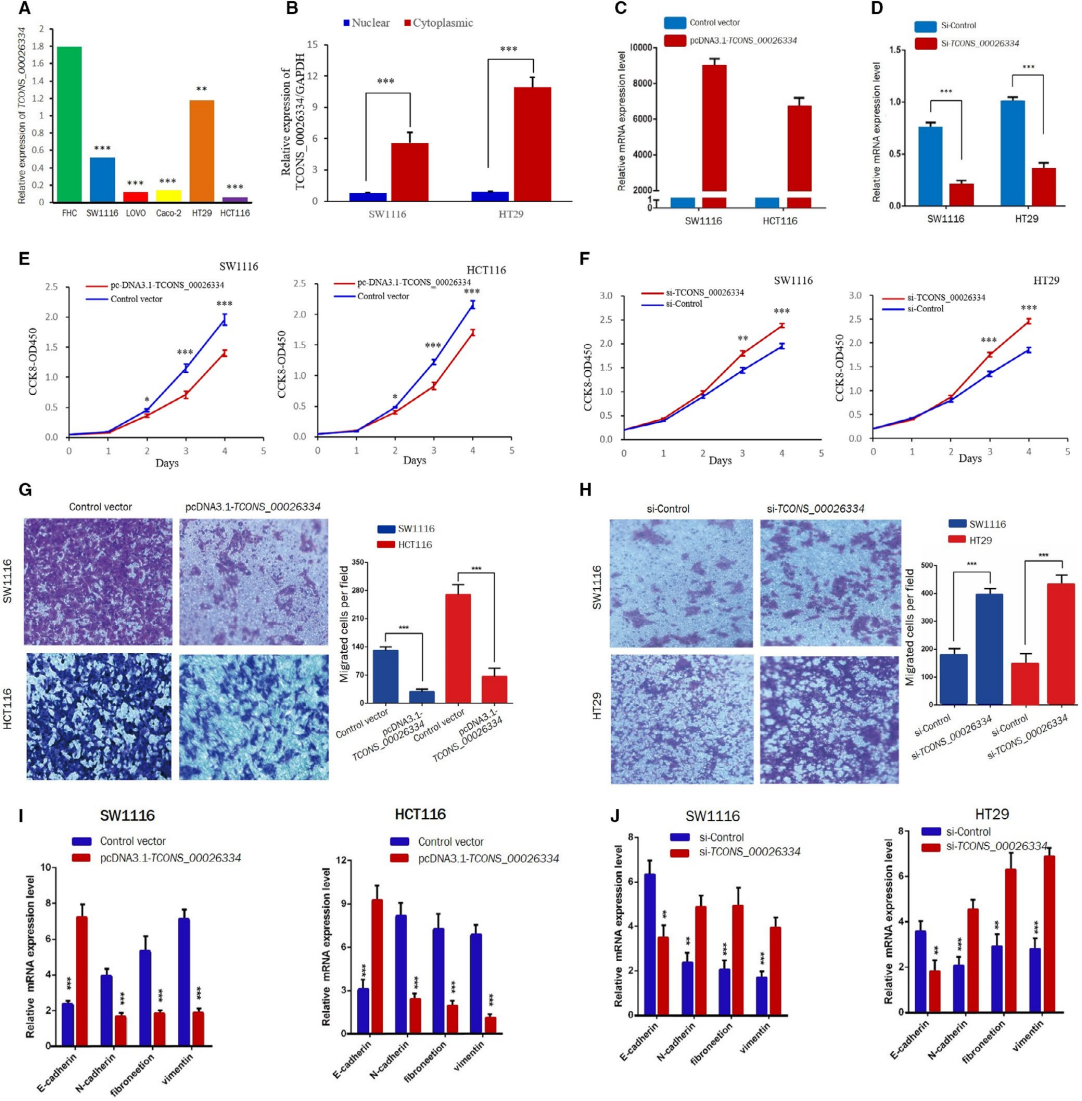
*TCONS_00026334* induces EMT reversal and inhibits aggressive phenotypes in CRC cells. (A) QRT‐PCR results of the expression of *TCONS_00026334* in different CRC cell lines. (B) QRT‐PCR identified TCONS_00026334 was mainly distributed in the cytoplasm and little in the nucleus in SW1116 and HT29 cells. (C, D) The efficiency of *TCONS_00026334* siRNA or overexpression was confirmed by qRT‐PCR in CRC cell lines. (E, F) A CCK‐8 assay was used to assess the proliferative ability of CRC cells after transfection with pcDNA3.1‐TCONS_00026334 or control vector (E) or TCONS_00026334 siRNA or control siRNA (F) for *TCONS_00026334*. (G, H) The migration and invasion abilities of CRC cells were assessed after overexpression (G) or silencing (H) of *TCONS_00026334* using Transwell assays. (I, J) QRT‐PCR indicating upregulation and downregulation of mRNA expression of epithelial marker E‐cadherin and mesenchymal markers Vimentin/N‐cadherin/Fibronectin, after overexpression and silencing of *TCONS_00026334* in different CRC cells. The data are presented as mean ± SD (*p < 0.05, **p < 0.01, ***p < 0.001; *n* = 3 independent experiments).

We next tested the biological functions of *TCONS_00026334* in the development of CRC by synthesizing and transfecting plasmids (pcDNA3.1‐TCONS_00026334) or siRNAs targeting *TCONS_00026334* to upregulate or downregulate *TCONS_00026334* expression. The qRT‐PCR results showed different expression levels of *TCONS_00026334* in SW1116, Lovo, Caco‐2, HT29, and HCT116 cells in comparison to that in FHC cells (Figure 2A). *TCONS_00026334* in SW1116 and HT29 cells was predominately distributed in the cytoplasm and little in the nucleus (Figure 2B). Subsequently, SW1116 and HCT116 cells were selected to be transduced with plasmids (pcDNA3.1‐TCONS_00026334) to upregulate *TCONS_00026334* expression levels (Figure 2C). Contrastingly, SW1116 and HT29 cells were transfected with the siRNA to downregulate *TCONS_00026334* expression. The efficiency of the plasmids and siRNA were confirmed using quantitative real‐time PCR (qPCR; Figure 2D).

To observe the proliferation abilities *in vitro* of CRC cells after overexpressing and knocked down of *TCONS_00026334*, the CCK8 assay was used. The results indicated that that transfection with pcDNA3.1‐TCONS_00026334 greatly inhibited the proliferative ability of SW1116 and HCT116 cells (Figure 2E). Conversely, knockdown of *TCONS_00026334* increased cell viability relative to that of the control group (Figure 2F).

Next, the effect of *TCONS_00026334* on the invasion capabilities of CRC cells was detected by Transwell assays. The invasion capabilities of SW1116 and HCT116 cells were inhibited following *TCONS_00026334* upregulation (Figure 2G), but strongly enhanced by depleting *TCONS_00026334* in HT29 and SW1116 cells (Figure 2H).

The initiation of epithelial‐mesenchyme transition (EMT) drives tumor invasion and metastasis. Consequently, we detected the levels of EMT markers: E‐cadherin, Fibronectin, N‐cadherin, and Vimentin. Interestingly, *TCONS_00026334* overexpression resulted in upregulation of E‐cadherin mRNAs expression while downregulation of mesenchymal markers mRNAs expression. *TCONS_00026334* silencing showed the opposite results (Figure 2I, J). Therefore, we concluded that *TCONS_00026334* might function as a tumor‐suppressive modulator to inhibit EMT progression in CRC.

### 3.3 LncRNA TCONS_00026334 could regulate the development of CRC by directly binding to miR‐548n

**Figure 3:**
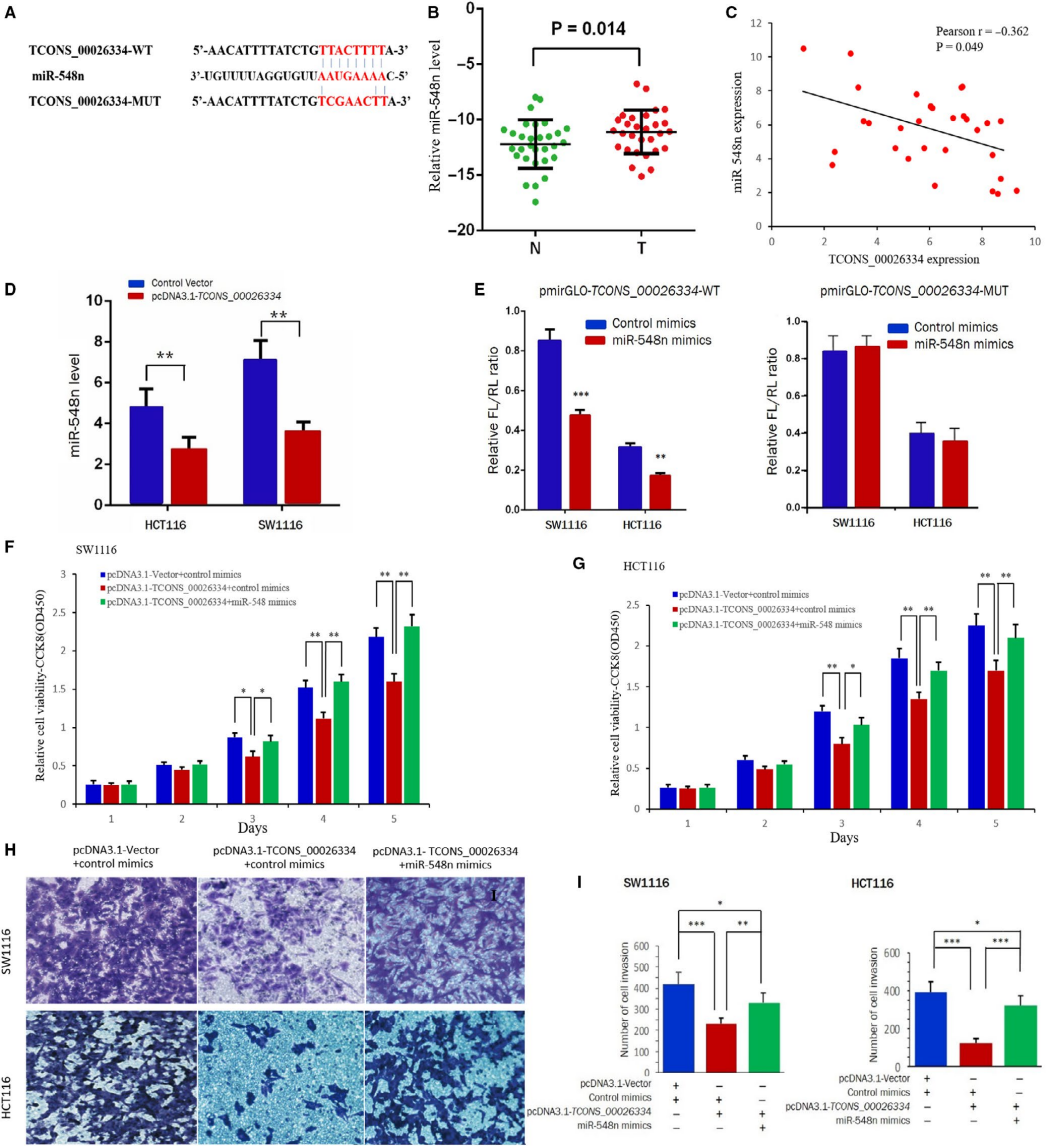
*TCONS_00026334* regulates miR‐548n expression negatively in CRC. (A) Bioinformatics software showed the predicted sequence of the *TCONS_00026334* binding site on the 3ʹ‐UTR of miR‐548n mRNA. (B) MiR‐548n expression was analyzed using qRT‐PCR in 30 paired CRC tissues and adjacent normal tissues. (C) Correlations between the expression levels of miR‐548n and *TCONS_00026334* in 30 paired CRC tissues. (D) The expression level of miR‐548n was detected in the presence of pcDNA3.1‐TCONS_00026334 using qRT‐PCR. (E) Luciferase reporter assay was performed after co‐transfection with miR‐548n and luciferase reporters containing *TCONS_00026334* or mutant transcripts in SW1116 and HCT116 cells. (F, G) The cell viabilities of SW1116 and HCT116 were detected after transfection with pcDNA3.1‐TCONS_00026334, or pcDNA3.1‐TCONS_00026334 and miR‐548n mimics, or pcDNA3.1‐control vector and control mimics using a CCK‐8 assay. (H, I) In the presence of pcDNA3.1‐TCONS_00026334, the efficiency of miR‐548n mimics on the invasive ability of CRC cells was detected using Transwell method. The data are shown as the mean ± SD (*p < 0.05, **p < 0.01, ***p < 0.001; *n* = 3 independent experiments).

Recent evidence shows that certain lncRNAs sponge the biological functions of miRNAs by acting as competing endogenous RNAs (ceRNAs).^14^, ^15^ Analysis using online software suggested the existence of miR‐548n binding sites in TCONS_00026334 (Figure 3A). Next, the expression pattern of miR‐548n was evaluated in patients with CRC. Compared with adjacent normal tissues, CRC tissues showed significantly higher expression levels of miR‐548n (Figure 3B). Furthermore, we found that the expression levels of TCONS_00026334 and miR‐548n correlated negatively (Figure 3C, *r* = −0.362, *p* = 0.049). Moreover, TCONS_00026334 overexpression decreased the level of miR‐548n in CRC cells (all *p* < 0.01; Figure 3D). To verify whether TCONS_00026334 is correlated with the upregulation of miR‐548n in CRC, we further confirmed the direct interactions between TCONS_00026334 and miR‐548n using luciferase reporter assays. Figure 3E shows that the overexpression of miR‐548n reduced the luciferase activities of the TCONS_00026334‐WT reporter but not the TCONS_00026334‐MUT reporter.

To further insight into the roles of *TCONS_00026334* and miR‐548n in regulating the carcinogenesis and invasion of CRC, we used miR‐548 mimics to counteract the inhibitory effects induced by pcDNA3.1‐TCONS_00026334 on the cell phenotypes in CRC. SW1116 and HCT116 cell lines were co‐transfected with a plasmid carrying the *TCONS_00026334* cDNA and miR‐548 mimics or pcDNA3.1 empty vector, and their functions were analyzed. In comparison with that in the control group, the overexpression of miR‐548 partially reversed the suppression effects on cell proliferation and invasion induced by *TCONS_00026334* overexpression in CRC cells (Figure 3F–I). These findings implied that *TCONS_00026334 *has a negative regulatory function via direct binding miR‐548n as a ceRNA in CRC.

### 3.4 TP53INP1 is the target of the TCONS_00026334/miR‐548n axis in CRC cells

**Figure 4:**
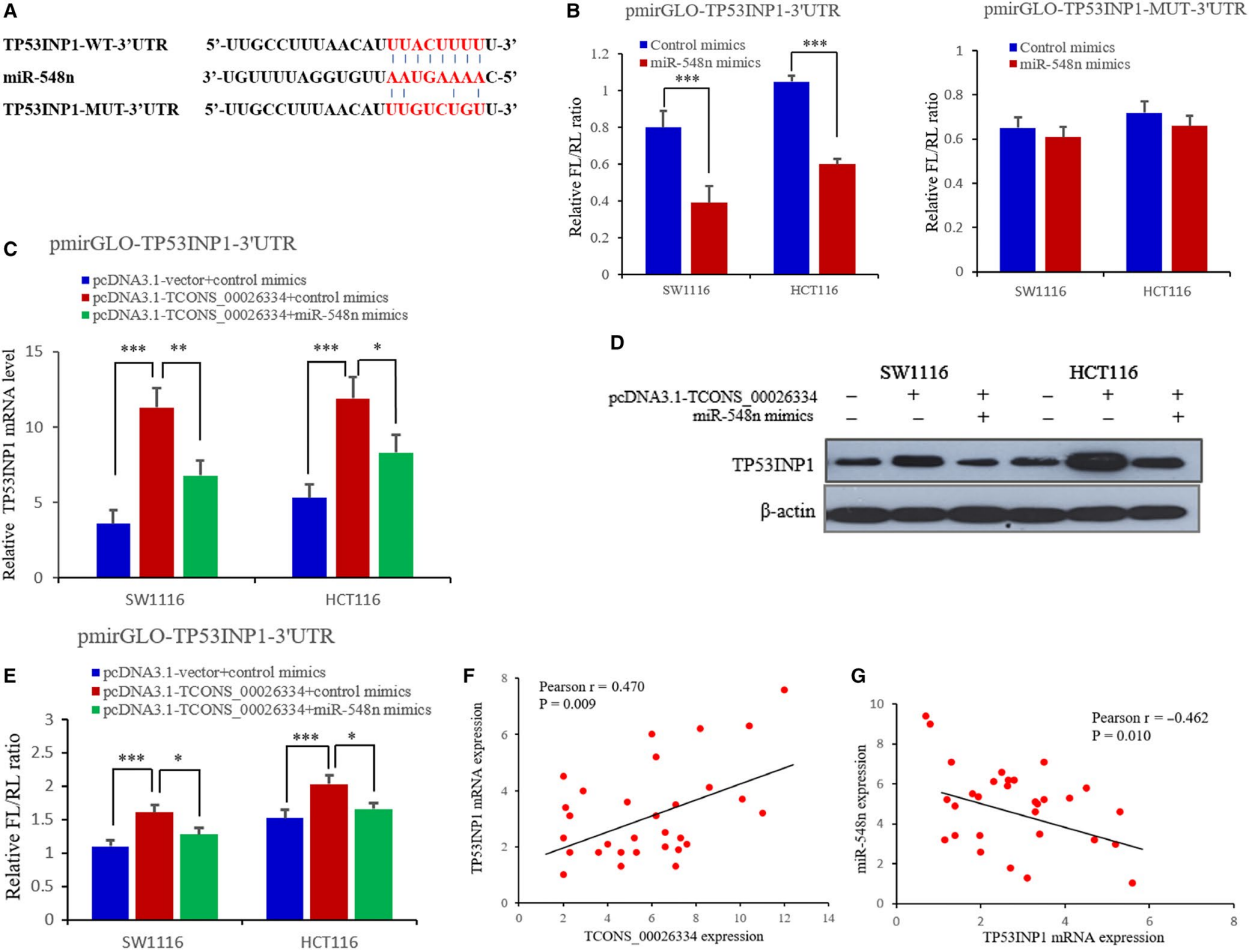
*TCONS_00026334* affects the expression of miR‐548n target *TP53INP1* in CRC cells. (A) Bioinformatics prediction of miR‐548n binding sites in the *TP53INP1* 3′‐UTR region. (B) Luciferase reporter assay was performed after co‐transfection of pmirGLO‐TP53INP1‐3' UTR or pmirGLO‐TP53INP1‐MUT‐3'UTR, in the attendance of miR‐548n mimics or negative controls. (C, D) The mRNA and protein level of *TP53INP1* were assayed after transfection with pcDNA3.1‐TCONS_00026334 or/and miR‐548n mimics in CRC cells. (E) The overexpression of TCONS_00026334 enhanced the luciferase intensity of pmirGLO‐*TP53INP1*‐3'‐UTR whereas miR‐548n mimics reduced this increased luciferase intensity. (F, G) Positive correlation between *TP53INP1* and *TCONS_00026334* (F) and negative correlation between *TP53INP1* and miR‐548n in 30 paired CRC specimens. (G). The data are shown as the mean ± SD (*p < 0.05, **p < 0.01, ***p < 0.001; *n* = 3 independent experiments).

Increasing evidence showed that the human *TP53INP1* plays the role of anti‐oncogene in CRC, and could be regulated by miRNAs as a critical target, including miR‐125b, miR‐524‐5p, and miR‐200a.^21^, ^22^, ^23^, ^24^ In the present study, we used the bioinformatics software to detect the potential downstream targets of mi548n. Finally, *TP53INP1* was screened out as a novel candidate, which has miR‐548n binding sites within the 3'‐UTR of its mRNA (Figure 4A). To further confirm the possibility that miR‐548n can directly bind to the predicted sites of *TP53INP1*, the *TP53INP1* 3'‐UTR fragment having the wild‐type or mutant binding sites was inserted into the downstream of luciferase reporter gene (Figure 4A). We found that the luciferase activity of pmirGLO‐TP53INP1‐3' UTR was reduced dramatically after treatment with miR‐548n mimics, but did not change in CRC cells transfected with pmirGLO‐TP53INP1‐MUT‐3'UTR (Figure 4B), this results indicated that miR‐548n could bind to the *TP53INP1* 3'‐UTR directly.

In order to determine whether *TCONS_00026334* suppresses CRC progression by modulating the expression of miR‐383 targets, Western blot and RT‐qPCR analysis were performed in CRC cells. We found that *TCONS_00026334* overexpression increased the *TP53INP1* level in SW1116 and HCT116 cells, whereas treatment with miR‐548n mimics abolished the increased *TP53INP1* expression mediated by pcDNA3.1‐*TCONS_00026334* (Figure 4C, D). Furthermore, luciferase reporter system was applied to detect TP53INP1 3'‐UTR intensity in CRC cells SW1116 and HCT116. The results indicated that the overexpression of *TCONS_00026334* enhanced the luciferase activity of pmirGLO‐ TP53INP1‐3'‐UTR, whereas miR‐548n mimics reduced this increased luciferase intensity (Figure 4E). At last, we found that there was a positive correlation between *TP53INP1* and *TCONS_00026334* (*r* = 0.470, *p* = 0.009), and a negative correlation between TP53INP1 and miR‐548n (*r* = −0.462, *p* = 0.010) in 30 paired CRC specimens (Figure 4F, G).

### 3.5 TCONS_00026334 inhibited CRC cell proliferation in vivo

**Figure 5:**
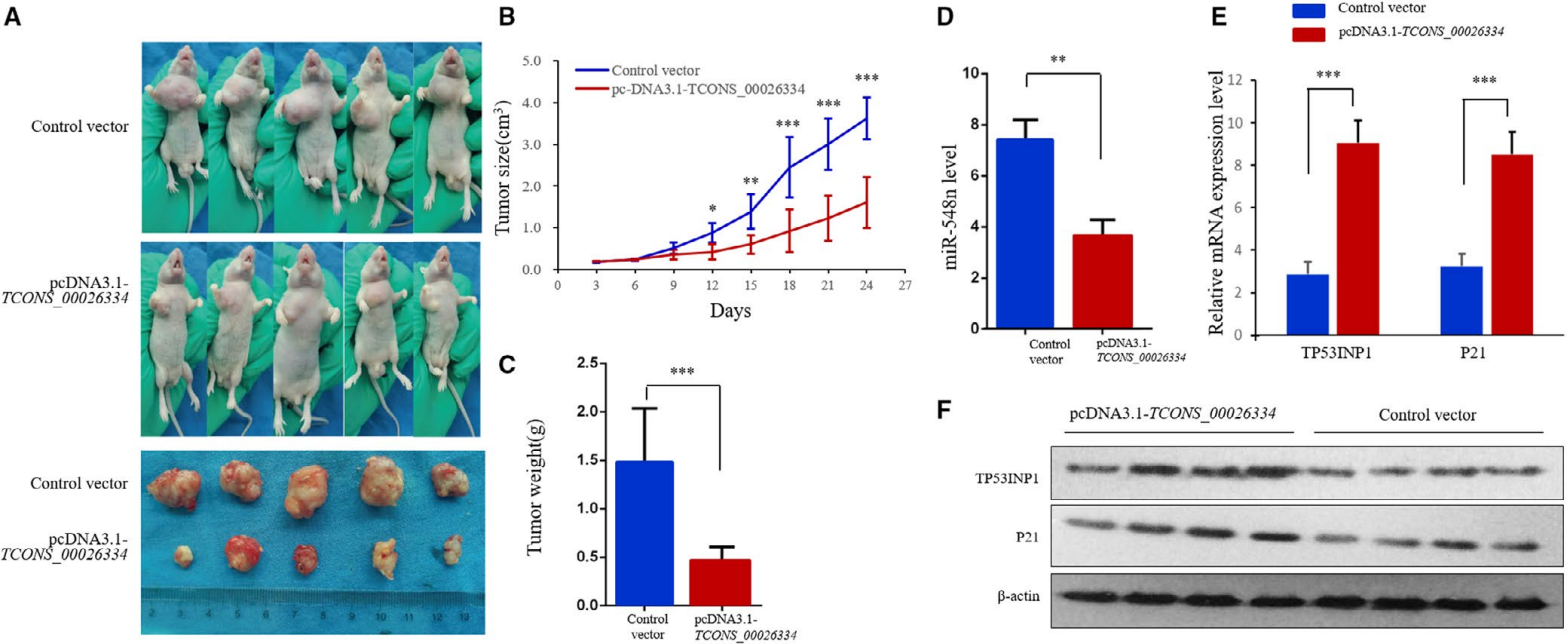
TCONS_00026334 overexpression suppresses tumor formation via downregulation of miR548n. (A–C) Tumor volume and weight of nude mice after injection of transfected SW1116 cells. **p < 0.01 versus the control group. ***p < 0.001 versus the control group. The data is presented as the mean ± SD. Repeated measurement ANOVA was used to compare the values at different time points; *N* = 5. The independent experiments were repeated three times. (D) The mRNA expression of miR‐548n after transfection with pcDNA3.1‐TCONS_00026334. (E, F) The mRNA expression (E) and protein expression levels (F) of TP53INP1 and P21 after transfection with pcDNA3.1‐TCONS_00026334.

To explore the *in vivo* tumorigenic effects of *TCONS_00026334* and miR‐548n, SW1116‐control vector cells or SW1116‐pcDNA3.1‐TCONS_00026334 cells were implanted into nude mice subcutaneously. Twenty‐five days later, the tumors were removed (Figure 5A). The tumor volume and weight, and tumoral miR‐548n expression were decreased significantly by *TCONS_00026334* overexpression (Figure 5B–D), whereas the *TP53INP1* and P21 expression increased obviously (Figure 5E, F). Collectively, these findings suggested that *TCONS_00026334* overexpression had the ability to restrain tumor growth *in vivo* via downregulating miR‐548n to increasing the expression of *TP53INP1*.

## 4 DISCUSSION

Mounting evidence indicates that CRC tumorigenesis, metastasis, and prognosis are associated with aberrant lncRNA expression. LncRNAs could act as tumor suppressors or oncogenes, for example *GAPLINC*, *NEAT1*, *HOTAIR*, and *LINC00265*.^11^, ^12^, ^25^, ^26^ In this study, we identified that the lncRNA *TCONS_00026334* was frequently downregulated in CRC tissues. Decreased *TCONS_00026334* levels were related to CRC progression and poorer prognosis of patients with CRC. *TCONS_00026334* overexpression inhibited the cell proliferation and invasion abilities of CRC significantly. Moreover, overexpression of *TCONS_00026334* could simultaneously upregulate the mRNA expression of E‐cadherin and downregulate the mRNA expression of mesenchymal markers, which could promote the invasion and migration of tumor cells, leading to cancer metastasis.^27^, ^28^ To sum up, these results identified that *TCONS_00026334* suppresses tumor progression and metastasis, and may represent a potential biomarker for CRC therapy.

As a novel lncRNA, the potential mechanism of action of *TCONS_00026334* remains largely unknown, unlike some other well‐studied lncRNAs.^25^, ^29^ Along with the role of transcriptional regulation, many lncRNAs modulate gene expression by serving as endogenous target mimics for miRNAs.^17^, ^22^, ^30^ Such as, *NEAT1* could regulate the expression of miR‐133b by acting as a ceRNA to influence cell viability and invasion in breast cancer cells.^31^ In the tumorigenesis of hepatocellular carcinoma (HCC), lncRNA *PTTG3P* sponges miR‐383, thereby upregulating the expression of miR‐383's target, Cyclin D1 (CCND1).^32^ Therefore, we investigated the molecular mechanism by which *TCONS_00026334* interacts with miRNAs in CRC. Bioinformatics analysis indicated potential interactions between *TCONS_00026334* and miR‐548n. MiR‐548n was upregulated in the tumor tissues of CRC and luciferase assays validated that miR‐548n directly binds to *TCONS_00026334*. This interaction affects cell proliferation and invasion of CRC. Overall, this study demonstrated that *TCONS_00026334* exerts its function as a ceRNA depending on the competitive binding of miR‐548n.

Recent studies showed that certain lncRNAs exert their functions partly by acting as ceRNAs and modulating the expression of miRNA targets ^18^, ^33^, ^34^. Accordingly, bioinformatics analyses were used to find the candidate targets of miR‐548n. Finally, *TP53INP1* was screened out as the direct target of miR‐548n. *TP53INP1* is located on chromosome 8q22 and is downregulated in multiple cancers, such as breast cancer, prostate cancer, gastric cancer, advanced HCC, and CRC.^35^, ^36^ Some recent studies also indicated that *TP53INP1*, as a tumor suppressor gene, could be regulated by miRNAs as a critical target, including miR‐125b, miR‐524‐5p, and miR‐200a.^22^, ^23^, ^24^ Our luciferase results showed that miR‐548n binds to the 3ʹ UTR of *TP53INP1* directly and *TCONS_00026334* could upregulate the mRNA and protein expression of *TP53INP1*, while miR‐548n mimics could abrogate the increased *TP53INP1* expression mediated by pcDNA3.1‐TCONS_00026334. In addition, luciferase reporter assay indicated that the overexpression of *TCONS_00026334* improved the luciferase intensity of pmirGLO‐TP53INP1‐3'‐UTR, whereas miR‐548n mimics reduced this increased luciferase intensity. These above results inferred that *TCONS_00026334* regulates the miR‐548n/*TP53INP1* feedback loop and inhibits the development of CRC by functioning as a ceRNA to sponge miR‐548n.

In summary, the results of this study identified the *TCONS_00026334* acts as an anti‐tumor and anti‐metastatic gene to inhibit CRC development by competitively binding miR‐548n, upregulating *TP53INP1* expression. Therefore, the *TCONS_00026334*–miR‐548n–*TP53INP1* network might represent a useful biomarker and promising target against CRC therapy.

## CONFLICT OF INTEREST

All authors have no conflicts of interest to declare.

## AUTHOR CONTRIBUTIONS

MMZ, ATX, YL, MZ, and ZHR conceived and designed the experiments; MMZ and YL performed the experiments; ATX, XTX, and YL analyzed the data; MMZ wrote the paper. All authors read and approved the final manuscript.

## Supporting information

Table S1‐S3

Supplementary Material

## Data Availability

The microarray dataset is being used in our laboratory for further studies, therefore it is not publicly available.
