# Supplementary material for: Long noncoding RNA TCONS_00026334 is involved in suppressing the progression of colorectal cancer by regulating miR‑548n/TP53INP1 signaling pathway
Source: Cancer Med. 2020 Sep 28;9(22):8639–49. doi: 10.1002/cam4.3473 (PMC7666722; doi:10.1002/cam4.3473)
Supplement: Supplementary file 1 — Table S1‐S3 [file CAM4-9-8639-s001.docx]

Supplementary material Table S1. Sequences of oligomers and primers applied in this study

| Gene name | Primer sequence (5′ to 3′) |
| --- | --- |
| TCONS_00026334 | Forward: TCGGTGCAGTGGTGATTTTG  Reverse: TTTGAGCCTGACACGAACTCT |
| E-cadherin | Forward: AGAACGCATTGCCACATACACTC  Reverse: CATTCTGATCGGTTACCGTGATC |
| N-cadherin | Forward: TGAGCCTGAAGCCAACCTTA  Reverse: AGGTCCCCTGGAGTTTTCTG |
| Vimentin | Forward: AGCTAACCAACGACAAAGCC  Reverse: TCCACTTTGCGTTCAAGGTC |
| Fibronectin | Forward: CAGTGGGAGACCTCGAGAAG  Reverse: TCCCTCGGAACATCAGAAAC |
| miR-548n | Forward: CAAAAGUAAUUGUGGAUUUUGU  Reverse: AAAAUCCACAAUUACUUUUGUU |
| TP53INP1 | Forward: GCACCCTTCAGTCTTTTCCTGTT  Reverse: GGAGAAAGCAGGAATCACTTGTATC |
| GAPDH | Forward: GCATTGCCCTCAACGACCAC  Reverse: CCACCACCCTGTTGCTGTAG |

Supplementary material Table S2. The association between *TCONS_00026334* levels and clinical characteristics of 86 patients with colorectal cancer

| Clinical characteristics | Total 86 | *TCONS_00026334* expression | | P value |
| --- | --- | --- | --- | --- |
|  |  | Low (n = 43, 50%) | High (n = 43, 50%) |  |
| Age |  |  |  |  |
| ≤ 65 years | 53 | 27 (50.94%) | 26 (49.06%) | 0.825 |
| > 65 years | 33 | 16 (48.48%) | 17 (51.52%) |  |
| Gender |  |  |  |  |
| Male | 47 | 22 (46.81%) | 25 (53.19%) | 0.516 |
| Female | 39 | 21 (53.85%) | 18 (46.15%) |  |
| Tumor size |  |  |  |  |
| ≤ 5 cm | 48 | 19 (39.58%) | 29 (60.42%) | **0.030** |
| > 5 cm | 38 | 24 (63.16%) | 14 (36.84%) |  |
| Tumor location |  |  |  |  |
| Rectum | 49 | 28 (57.14%) | 21 (42.86%) | 0.127 |
| Colon | 37 | 15 (40.54%) | 22 (59.46%) |  |
| Serum CEA |  |  |  |  |
| ≤ 5 ng/ml | 40 | 18 (45.00%) | 22 (55.00%) | **0.018** |
| > 5 ng/ml | 46 | 29 (63.04%) | 17 (36.96%) |  |
| Distant liver metastasis |  |  |  |  |
| M0 | 69 | 30 (43.48%) | 39 (56.52%) | **0.015** |
| M1 | 17 | 13 (76.47%) | 4 (23.53%) |  |
| Clinical stage |  |  |  |  |
| I- II | 40 | 13 (32.50%) | 27 (67.50%) | **0.014** |
| III-Ⅳ | 46 | 30 (65.22%) | 16 (34.78%) |  |

Supplementary material Table S3. Univariable and multivariable analyses of prognostic factors for overall survival in 86 patients with colorectal cancer

| Prognostic factors | Univariable analysis | | | | Multivariable analysis | | | |
| --- | --- | --- | --- | --- | --- | --- | --- | --- |
|  | HR | 95% CI | P value |  |  | HR | 95% CI | P value |
| **TCONS_00026334 expression** (low *vs*. high) | 0.394 | 0.217–0.716 | **0.002** | | 0.419 | | 0.218–0.806 | **0.009** |
| **Age**  (≤ 65 *vs*. > 65) | 1.044 | 0.555–1.963 | 0.895 | | - | | - | - |
| **Gender**  (male *v*s. female) | 0.909 | 0.511–1.616 | 0.744 | | - | | - | - |
| **Tumor size**  (≤ 5 cm vs. > 5 cm) | 1.648 | 0.928–2.926 | 0.089 | | - | | - | **-** |
| **Tumor location**  (rectum *v*s. colon) | 0.848 | 0.473–1.523 | 0.582 | | - | | - | - |
| **Serum CEA**  (> 5 ng/ml *vs*. ≤ 5 ng/m*l*) | 1.944 | 1.092–3.458 | **0.024** | | 1.129 | | 0.580–2.196 | 0.721 |
| **Distant liver metastasis**  (yes *vs*. no) | 5.769 | 2.836–11.738 | **0.001** | | 4.513 | | 1.431–14.232 | **0.010** |
| **Clinical stage**  (I *vs.* II *vs.* III *vs.* IV) | 1.968 | 1.375–2.816 | **0.031** | | 0.975 | | 0.565–1.682 | 0.928 |
