# Supplementary material for: Long noncoding RNA TCONS_00026334 is involved in suppressing the progression of colorectal cancer by regulating miR‑548n/TP53INP1 signaling pathway
Source: Cancer Med. 2020 Sep 28;9(22):8639–49. doi: 10.1002/cam4.3473 (PMC7666722; doi:10.1002/cam4.3473)
Supplement: Supplementary file 2 — Supplementary Material [file CAM4-9-8639-s002.docx]

**TCONS_00026334**

<http://genome.ucsc.edu/cgi-bin/hgc?hgsid=750084857_deUV9p2w0oB3maVaCmSN3z69aqPe&c=chr18&l=54892643&r=54895106&o=54892643&t=54895106&g=lincRNAsTranscripts&i=TCONS_00026334>

**Position:** [chr18:52559875-52562337](http://genome.ucsc.edu/cgi-bin/hgTracks?hgsid=870524315_sIZPGrftPy88vJTLRGPHhOFzgeCi&db=hg19&position=chr18%3A52559875-52562337)
**Band:** 18q21.2
**Genomic Size:** 2463
**Strand:** +

**Sequence**

cgccaagagttgagcctgtgggtctctccataagagttttaaaactcttg

ccagttaccactttatccaatttgctatcattttcgtattatcagctatc

gccctgtaaaatattcaaaactagctatttctaaagtaaacattttatct

gttacttttaaccagataggtgtctttgtcatccttctactataaattgt

tctttgccaacctgtacaggtagatgaaccaggcgagagttttaatcagc

cttttcttgtcccctttgtaagaaagagatgcttgccatagagaaggaca

tgagtacattaaaaataatttaatagccacaatatgatgttctttaagct

gcaaattgagtacactgggaatcaacaaatttgatgaagcctgtctgtct

cttcaccagtggagtgagtgcagcagttagaaagagaagcaatattgtgc

aactggtgcagtggtgattttgttttcaaatgctccttgtgaaaacacct

agtgttgtagaaaggaaagtggccagaaagaacaacttgggaccatgagt

aggtcattaaatagcttagtgatttatcctcatatagggcttataaaccc

tgtatgtgtttatatgtgcttcacagagttcgtgtcaggctcaaaggaga

tatgtataagaaagtggtttgtaaattatgttccatttcataaatagaca

ctattcacaaactaaaatctaataaaaaaccacagttgtaatttaaactg

cttgatataaaaagaggtatcatagcagggaaaacacactaattttcata

cagtagaggtattgaaaactgaaaatgggaaggcaacttgaagtcattgt

atttgattgaaaatgtttaatacatctcattattgacaaaatatgtcatc

ttgtatttatttcaaggaaaccaatgaattctaggtagtatattacaagt

tggtcaaaatattccatgtacaaatagggcttctgtgtccatagccttgt

aagagatactgattgtatctgaaattattttttaaaaaaa
